# A Cross‐Sectional, Mixed‐Methods Analysis of Affective Symptoms and Quality‐of‐Life in Persons Experiencing Subjective Cognitive Decline

**DOI:** 10.1002/alz70860_105947

**Published:** 2025-12-23

**Authors:** Rebecca Ann‐Maria Watry, Dereck L. Salisbury, Sarah Hoffman, Joseph Gaugler

**Affiliations:** ^1^ University of Minnesota, Minneapolis, MN, USA

## Abstract

**Background:**

Affective symptoms, (i.e., anxiety and depressive) in the presence of subjective cognitive decline (SCD), increase the risk of Alzheimer's disease (AD) dementia and decrease quality‐of‐life (QoL). The hazard ratio of dementia for SCD with affective symptoms is significantly worse compared to SCD alone. Although multiple causal hypotheses have been explored, the symptom and QoL relationships remain poorly understood. This literature gap is greater for qualitative and mixed‐methods research. Furthermore, there is a gap in exploring the relationships between SCD symptoms with affective symptoms and QoL. We anticipate that an explanatory sequential analysis will elucidate themes and patterns from the participants' perspectives.

**Methods:**

The SCD‐I working group criteria for SCD+ (more likely to have preclinical AD and progress to dementia; operationalized as >3 SCD+ features) guided the secondary data analysis of the Exergames Study (*N* = 38 older adults with SCD). Participants were divided into SCD+ (*n* = 24) and non‐SCD+ (*n* = 14) groups. Quantitative data were analyzed for nine relationships. Supportive and divergent themes and patterns from a cross‐thematic analysis will be compared to the quantitative data in a mixed‐methods analysis.

**Results:**

Enrollment spanned 2019‐2021. The mean age was 74.6 (7.5) years old and 69% were female. Emergent themes and patterns will explain the following quantitative results: 1) Significant correlations were found between anxiety and depressive symptoms (*N* = 38, r = 0.44, *p* <0.01), 2) QoL had significantly negative correlations with both depressive and anxiety symptoms (*N* = 38; r = ‐0.49, *p* <0.01; r = 0.38, *p* = 0.03, respectively), 3) depressive and anxiety symptoms were significantly correlated with psychological QoL (*N* = 38; r = ‐0.53, *p* <0.01; r = ‐0.57, *p* <0.01, respectively), 4) In SCD+ (*n* = 24) significant correlations were found between SCD symptoms and QoL with depressive symptoms, and between depressive and anxiety symptoms with psychological QoL (*r* = 0.41, *p* = 0.05; r = ‐0.58, *p* <0.01; r = 0.54, *p* <0.01; r = ‐0.65, *p* <0.01; respectively).

**Conclusion:**

A mixed‐methods analysis is necessary to explain the relationships found and those not found in SCD and SCD+. A greater understanding of the relationships in SCD and in SCD+ from a mixed‐methods perspective will add to the research knowledge base and guide future interventional non‐pharmacological research.

